# Characterization of the Plasma Lipidome in Dairy Cattle Transitioning from Gestation to Lactation: Identifying Novel Biomarkers of Metabolic Impairment

**DOI:** 10.3390/metabo11050290

**Published:** 2021-04-30

**Authors:** Jorge Eduardo Rico, Sina Saed Samii, Yu Zang, Pragney Deme, Norman J. Haughey, Ester Grilli, Joseph W. McFadden

**Affiliations:** 1 Department of Animal Science, Cornell University, Ithaca, NY 14853, USA; jerico@umd.edu; 2 Department of Animal and Avian Sciences, University of Maryland, College Park, MD 20742, USA; 3 Division of Animal and Nutritional Sciences, West Virginia University, Morgantown, WV 26505, USA; ssamii@witmersfeed.com (S.S.S.); zang@whminer.com (Y.Z.); 4 Department of Neurology, Johns Hopkins University School of Medicine, Baltimore, MD 21287, USA; pdeme1@jhmi.edu (P.D.); nhaughe1@jhmi.edu (N.J.H.); 5 Department of Veterinary Medical Sciences, University of Bologna, 40064 Bologna, Italy; eg@vetagro.com

**Keywords:** biomarker, cow health, peripartum, lipidomics

## Abstract

The discovery of novel biomarkers for peripartal diseases in dairy cows can improve our understanding of normal and dysfunctional metabolism, and lead to nutritional interventions that improve health and milk production. Our objectives were to characterize the plasma lipidome and identify metabolites associated with common markers of metabolic disease in peripartal dairy cattle. Multiparous Holstein cows (*n* = 27) were enrolled 30 d prior to expected parturition. Blood and liver samples were routinely collected through to d 14 postpartum. Untargeted lipidomics was performed using quadrupole time-of-flight mass spectrometry. Based on postpartum measures, cows were categorized into low or high total fatty acid area under the curve (total FAAUC; d 1–14 postpartum; 4915 ± 1369 vs. 12,501 ± 2761 (μmol/L × 14 d); *n* = 18), β-hydroxybutyrate AUC (BHBAAUC; d 1–14 postpartum; 4583 ± 459 vs. 7901 ± 1206 (μmol/L × 14 d); *n* = 18), or liver lipid content (d 5 and 14 postpartum; 5 ± 1 vs. 12 ± 2% of wet weight; *n* = 18). Cows displayed decreases in plasma triacylglycerols and monoalkyl-diacylglycerols, and the majority of phospholipids reached a nadir at parturition. Phosphatidylcholines (PC) 32:3, 35:5, and 37:5 were specific for high total FAAUC, PC 31:3, 32:3, 35:5, and 37:5 were specific for high BHBAAUC, and PC 31:2, 31:3, and 32:3 were specific for high liver lipid content. PC 32:3 was specific for elevated total FA, BHBA, and liver lipid content. Lipidomics revealed a dynamic peripartal lipidome remodeling, and lipid markers associated with elevated total FA, BHBA, and liver lipid content. The effectiveness of nutrition to impact these lipid biomarkers for preventing excess lipolysis and fatty liver warrants evaluation.

## 1. Introduction

In high-producing dairy cows, the prevalence of metabolic disease is elevated during the transition from gestation to lactation and estimated to affect ~40% of the dairy cow population [1]. Metabolic diseases, such as ketosis, fatty liver, mastitis, metritis, milk fever, and displaced abomasum, develop in association with the peripartal onset of negative energy balance (NEB), where feed consumption is commonly insufficient to meet the increased nutritional demands of lactation [2]. Metabolic disease compromises milk production and reproductive performance, and increases the economic cost of dairy production [3,4,5]. The early detection or prevention of metabolic disease has the potential to improve cow health during the peripartum, as well as sustain milk production, fertility, and profitability [6,7,8].

At the present time, circulating total fatty acids (FA) and β-hydroxybutyrate (BHBA) are industry standard biomarkers for the detection of metabolic impairment during the peripartal period. Laboratory and cow-side tests have been developed to monitor circulating total FA and BHBA status. The alarm threshold levels for poor reproductive performance and milk production for prepartum total FA and BHBA are 270 and 550 µmol/L, respectively, and a postpartum total FA of 600 µmol/L [9,10]. Unfortunately, total FA and BHBA testing are of limited value in that (a) these metabolite thresholds commonly lack in predictive value, as they often provide a late indication of ongoing metabolic dysfunction, (b) FA and BHBA do not distinguish between clinically healthy and sick cows considering that some animals experience elevations in total FA and/or BHBA but sustain normal milk production and health, and (c) beyond their relations to the severity of NEB and lipolysis, these biomarkers provide only a limited picture of the global changes in metabolism that cows undergo during the peripartum. In this context, we consider that the search for alternative biomarkers is warranted.

Lipidomics is a systems biology approach that emerged in the field of life sciences to investigate metabolite alterations in biological samples [11,12,13]. The application of lipidomics to identify biomarkers of metabolic disease in non-ruminants has been extensively employed [13,14,15]. However, only a limited number of studies have employed this analytical technology to better understand dairy cow health and performance [7,16,17]. Recent work has identified several phosphatidylcholines (PC) as biological indicators of postpartum metabolic disease [7]. Phosphatidylcholine is indeed a promising lipid class in the study of metabolic dysfunction, particularly in relation to the development of fatty liver, because of its role in the synthesis of hepatic lipoproteins responsible for the export of triacylglycerols (TAG; [18]). For instance, unsaturated and saturated PC ranging from 30 to 42 carbons appear to distinguish between healthy cows or those manifesting a clinical disease [16]. Despite these insights, our ability to generalize and extrapolate these types of data for widespread application is currently limited by factors such as small sample sizes, as well by a lack of standardization in the metabolites and diseases studied. The comprehensive characterization of the bovine lipidome provides the opportunity to improve our understanding of cow metabolism and its relationship with well-established phenotypes of metabolic impairment during the peripartum (i.e., elevated blood total FA, BHBA, and liver lipid content). Therefore, our objectives were two-fold: (1) to characterize the plasma lipidome of Holstein dairy cows transitioning from gestation to lactation, and (2) to identify biomarkers related to elevations in plasma total FA, BHBA, and liver lipid accumulation.

## 2. Results

### 2.1. Animal Parameters and Metabolic Status during Peripartum

**Figure 1:**
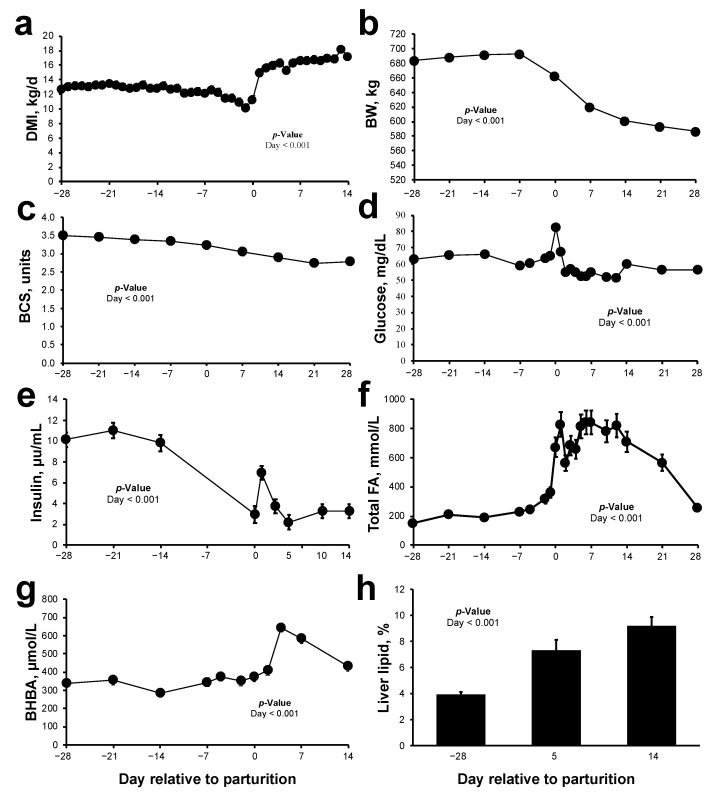
Changes in dry matter intake (DMI), body weight (BW) and body condition score (BCS), plasma metabolite concentrations and liver lipid content, in multiparous Holstein dairy cows (*n* = 27) transitioning from gestation to lactation. (**a**) DMI, (**b**) BW, (**c**) BCS; plasma concentrations of (**d**) glucose, (**e**) insulin (**f**) total fatty acids (FA) (**g**) β-hydroxybutyrate (BHBA), and (**h**) liver lipid content (%). Data were analyzed using a mixed effects model and presented as least squares means and their standard errors.

The transition from gestation to lactation was accompanied by suppressed dry matter intake (DMI; Figure 1a; *p* < 0.001), as well as progressive BW and BCS loss (Figure 1b,c; *p* < 0.001). Plasma glucose concentrations peaked at parturition and decreased thereafter (Figure 1d; *p* < 0.001). Plasma insulin concentrations decreased around parturition, coinciding with an elevation in plasma total FA, BHBA, and a progressive accumulation of liver lipid content (Figure 1e–h; *p* < 0.001). 

### 2.2. Plasma Lipidome Remodeling during the Peripartum

**Figure 2:**
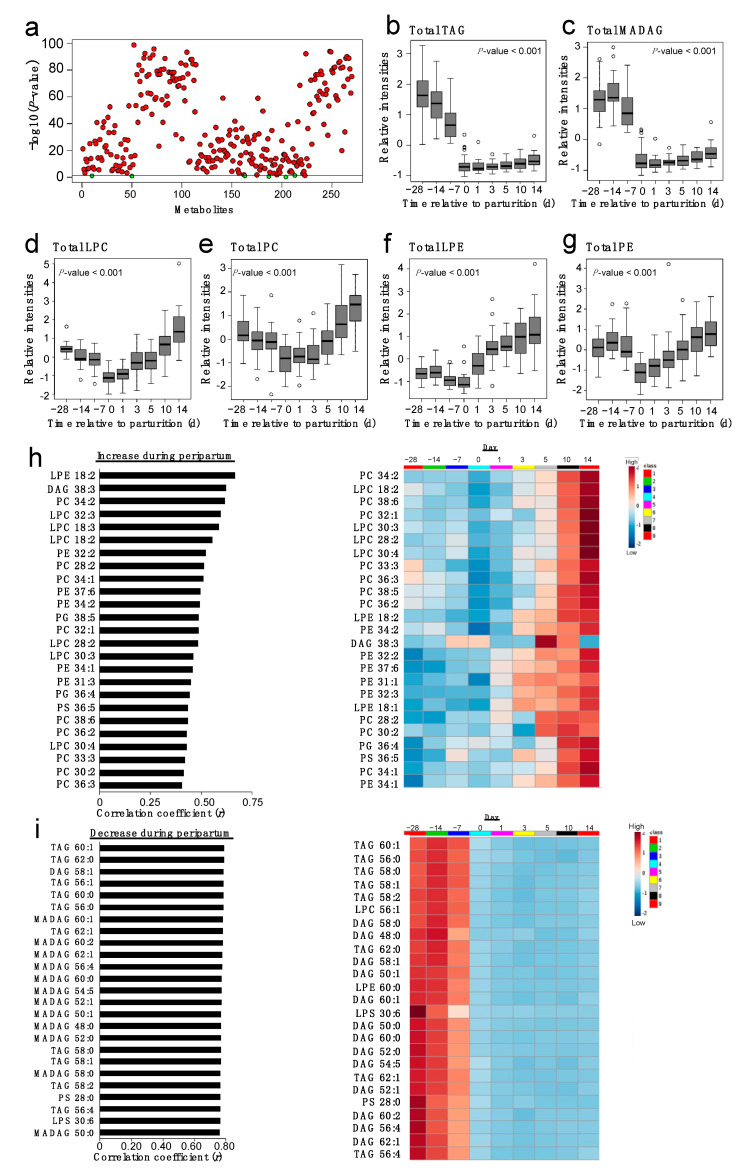
Changes in neutral lipids and phospholipids during the transition from gestation to lactation. (**a**) ANOVA of auto-scaled and normalized lipids; significant changes indicated by red circles for −log10-scaled raw *p*-values across the Y axis. Normalized, auto-scaled data reflect the sum of (**b**) 49 TAG (C38–C64), (**c**) 64 MADAG (C40–C62), (**d**) 29 lysophosphatidylcholine (LPC; C14–C30), (**e**) 69 phosphatidylcholine (PC; C24–C42), (**f**) 5 lysophosphatidylethanolamine (LPE; C18–C26), and (**g**) 38 phosphatidylethanolamine (PE; C26–C45) species measured in plasma. (**h**) Top 25 lipid species that increased or (**i**) decreased during postpartm. The panels represent data from multiparous Holstein dairy cows (*n* = 27) at nine time points spanning the peripartum (d −28 to d 14). For visualization purposes, the heat map represents log-transformed, auto-scaled data to showcase concentrations in a gradient as high (red) or low (blue). Lipidomics data were obtained using quadrupole time-of-flight mass spectrometry.

TripleTOF identified 301 plasma lipids, including 163 phospholipids (PC, phosphatidylethanolamine (PE), phosphatidylserine (PS), phosphatidylglycerol (PG), lysophosphatidylcholine (LPC), lysophosphatidylglycerol (LPG), lysophosphatidylethanolamine (LPE), lysophosphatidylserine (LPS)), 130 fatty acylglycerols (TAG, diacylglycerol (DAG), and monoalkyl-diacylglycerol (MADAG)) and 8 cholesteryl esters (CE). Dynamic changes were observed across lipid classes during the transition from gestation to lactation (Figure 2). ANOVA revealed changes in several lipid metabolites (FDR < 0.05; Figure 2a). Total TAG and MADAG decreased at parturition and remained low during postpartum (Figure 2b,c,i; *p* < 0.01), while total LPC, PC, LPE, and PE reached their nadir at parturition and progressively increased during postpartum (Figure 2d–h). Time-related changes were also observed for total CE, DAG, LPS, PS, LPG, and PG, as presented in Figure S1 (Supplementary Materials). Total CE, DAG and PG decreased around parturition and progressively increased postpartum (*p* < 0.001), while total LPS and PS decreased sharply at parturition and remained low during the postpartum evaluation period (*p* < 0.001). 

Our HPLC/TripleTOF MS data were loaded for PLS-DA analyses to investigate the global lipidome of plasma samples from multiparous Holstein dairy cows during the transition period. A two-dimensional, partial least squares discriminant analysis model (PLS-DA) model was applied to identify a subset of variables that distinguishes peripartal time points (d −28 to d 14, relative to parturition), and a variable importance of projection (VIP) score of > 1 based on Component 1 (explaining 40% of variation) of the PLS-DA model was used to determine the relative contribution of lipid species to discriminate between time points (Figure S2, Supplementary Materials). PLS-DA score plots illustrate a significant separation across all time points (Figure S2a, Supplementary Materials). Variance for Component 1 was explained by temporal changes in TAG, MADAG, PS, and LPS (Figure S2b, Supplementary Materials). Specifically, we observed a dominant lipid pattern marked by a time-dependent reduction in several TAG (e.g., TAG 56:4; 58:1, 60:1), MADAG (e.g., MADAG 56:4, 58:1, 60:1) PS 28:0; and LPS 30:6 during the peripartum. Similarly, Component 2, which explained 19% of the variance (Figure S2a, Supplementary Materials), was characterized by a progressive reduction in several plasma TAG (e.g., TAG 60:1 and 62:0), MADAG (e.g., MADAG 60:1 and 60:2), PS 28:0; and LPS 30:6, although specific elevations in LPE 18:2, PC 34:2, LPC 18:2, and DAG 38:3 were observed (Figure S2c, Supplementary Materials). 

Random forest analysis was performed to identify key metabolites that discriminate between time points during the transition period (nine datapoints from pre to postpartum), and which display a high prediction accuracy upon bootstrap sampling. Random forest classification was obtained following the prediction of the classification error rates (Figure S3a, Supplementary Materials). Important metabolites that differentiate peripartal time points were obtained by ranking their mean contributions to classification accuracy when permuted (Figure S3b, Supplementary Materials). As cows approached parturition, TAG 38:2 and 58:1, LPS 30:6, and MADAG 58:1, 46:0, 54:1, and 60:7 declined and remained low until d 14, whereas DAG 38:3, LPE 18:2, LPC 16:0 and 18:2, PG 36:2, and PC 36:2 and 34:2 increased after parturition. 

**Figure 3:**
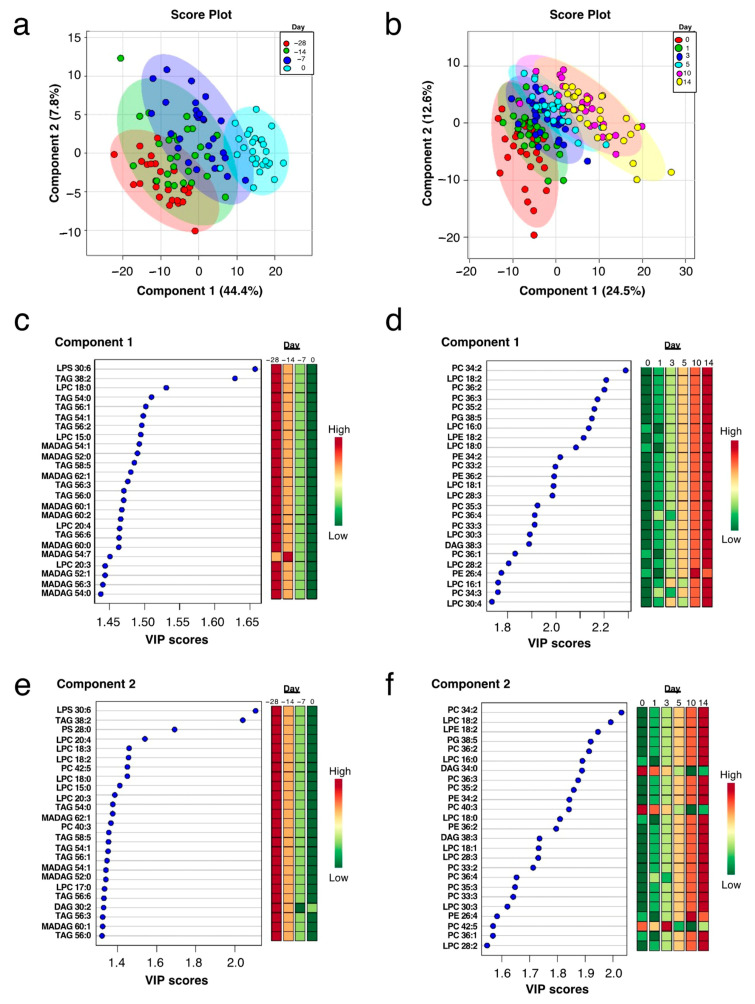
Plasma lipids lysophosphatidylserine (LPS) and triacylglycerol (TAG) prepartum and phosphatidylcholine (PC) and lysophosphatidylcholine (LPC) postpartum decrease drastically. (**a**) Two-dimensional partial least squares discriminant (PLS-DA) score plot prepartum, (**b**) two-dimensional PLS-DA score plot postpartum, (**c**) VIP scores analysis based on Component 1 of the PLS-DA prepartum, (**d**) VIP scores analysis based on Component 1 of the PLS-DA postpartum, (**e**) VIP scores analysis based on Component 2 of the PLS-DA prepartum, and (**f**) VIP scores analysis based on Component 2 of the PLS-DA postpartum used to rank the relative contribution of metabolites to the variance between time points. Prepartum variance for Component 1 is explained by changes in LPS, TAG, LPC, and monoalkyl-diacylglycerol (MADAG). Postpartum variance for Component 1 is explained by changes in PC, LPC, phosphatidylglycerol (PG), lysophosphatidylethanolamine (LPE), phosphatidylethanolamine (PE), and diacylglycerol (DAG). Normalized, auto-scaled data are representative of plasma collected from multiparous Holstein dairy cows (*n* = 27) prior to morning feeding at four time points spanning the prepartum and five time points spanning the postpartum. Lipidomics data were obtained using quadrupole time-of-flight mass spectrometry.

Prepartum data were isolated, and a two-dimensional PLS-DA model was used to identify a subset of variables that distinguishes between prepartum time points (Figure 3a). Plasma lipids LPS, TAG, LPC, and MADAG decreased progressively as parturition approached. Prepartum variance was explained by changes in several LPS, TAG, LPC, and MADAG (Figure 3c,e), with specific reductions in lipid metabolites observed consistently across component 1 and 2 (e.g., LPS 30:6; TAG 38:2, 54:0, 56:1, 54:1, 56:2, 56:3, 56:0, and 56:6; LPC 18:0, 15:0, 20:4, and 20:3; MADAG 54:1, 52:0, 62:1, 60:1, and 56:3). 

Postpartum data were isolated, and a two-dimensional PLS-DA model was used to identify metabolites that distinguish time points (Figure 3b). Postpartum variance for Components 1 and 2 was explained by changes in PC, LPC, PG, LPE, PE, and DAG, most of which progressively increased during the postpartum period (Figure 3d,f). Specific postpartum increases in PC 34:2, 36:2, 36:3, 35:2, 33:2, 35:3, 36:4, 33:3, and 36:1, LPC 18:2, 16:0, 18:0, 18:1, 28:3, 30:3, and 28:2, PG 38:5, LPE 18:2, PE 34:2, 36:2, and 26:4, and DAG 38:3 across Components 1 and 2 were observed (Figure 3d,f).

### 2.3. Correlations of PC and TAG Species

Due to the characterized relationship between hepatic PC synthesis and very low-density lipoprotein (VLDL)-TAG export, we explored the relationship between circulating PC and TAG using Pearson’s correlation coefficient analysis (Figure S4, Supplementary Materials). Heat map of *r*-values revealed that 69 PC (C24–C42) were correlated with 49 TAG (C38–C64). Overall, plasma levels of PC species were positively or negatively correlated with the plasma levels of TAG, depending on fatty acyl chain length of PC species.

### 2.4. Identification of Biomarkers Associated with Total FA, BHBA, and Liver Lipid

**Figure 4:**
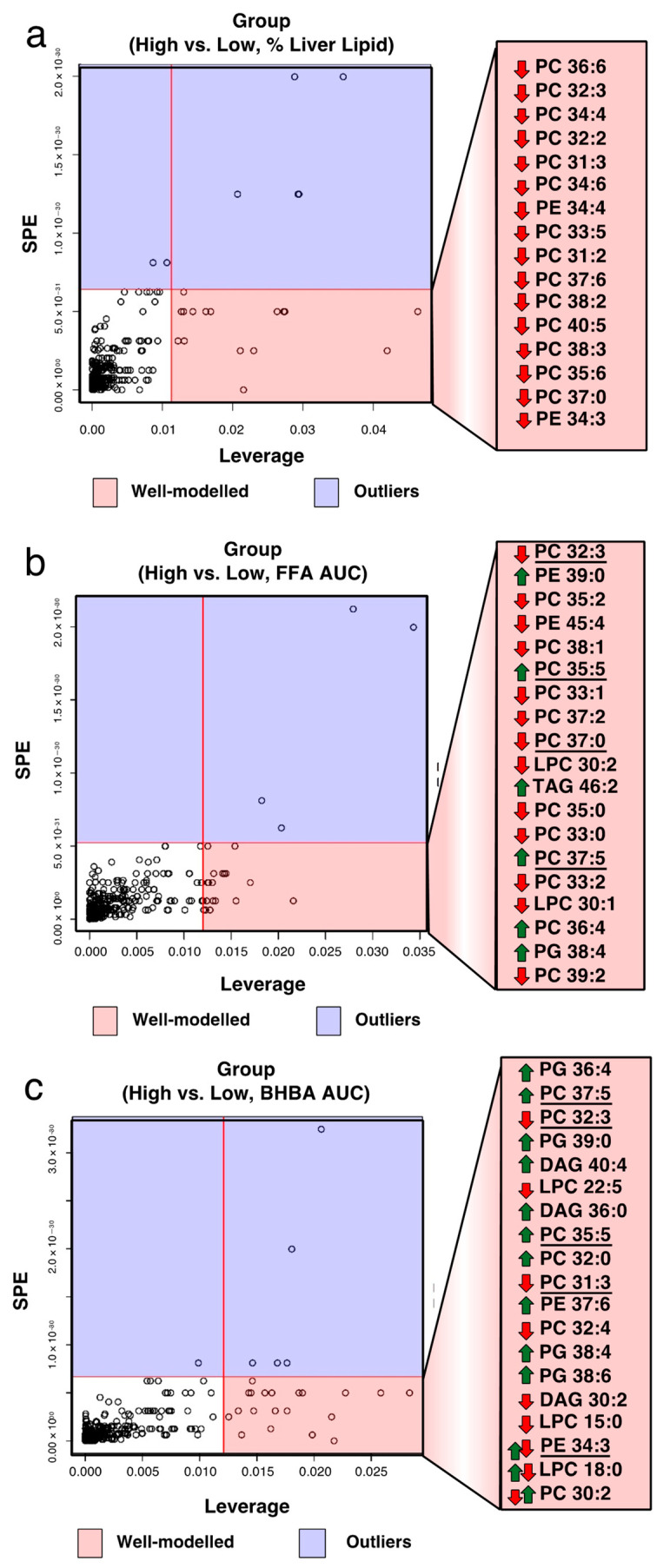
Biomarkers of peripartal liver lipid accumulation, circulating fatty acids (FA), and ketones. Suppressed plasma phosphatidylcholine (PC) levels are associated with fatty liver, high total FA, and BHBA area under the curve (AUC) concentrations in periparturient Holstein dairy cows. Leverage/squared prediction error (SPE) plot of 301 complex lipid and their relationship with (**a**) hepatic lipid accumulation, (**b**) elevated Total FA_AUC_ concentrations, and (**c**) elevated BHBA_AUC_ concentrations. Normalized, auto-scaled data represent data collected from periparturient Holstein dairy cows categorized into low (*n* = 9) or high (*n* = 9) mean (d 5 and 14 postpartum) liver lipid content (5 ± 1 vs. 12 ± 2% of wet weight, respectively), low (*n* = 9) or high (*n* = 9) Total FA_AUC_ (d 1–14 postpartum; 4915 ± 1369 vs. 12,501 ± 2761 (µmol/L × 14 d)), and low (*n* = 9) or high (*n* = 9) BHBA_AUC_ (d 1–14 postpartum; 4583 ± 459 vs. 7901 ± 1206 (µmol/L × 14 d)). Metabolites in the red area have high loadings and follow the expression pattern of the submodel (i.e., data indicate that out of 301 metabolites, the suppression of specific PC levels are most associated with fatty liver disease). PE = phosphatidylethanolamine. Lipidomics data were obtained using quadrupole time-of-flight mass spectrometry.

Based on postpartum metabolic health status, cows were categorized into low or high total FAAUC (d 1–14 pp; 4915 ± 1369 vs. 12,501 ± 2761 (µmol/L × 14 d)), BHBAAUC (d 1–14 pp; 4583 ± 459 vs. 7901 ± 1206 (µmol/L × 14 d)), or mean liver lipid content (d 5 and 14 pp; 5 ± 1 vs. 12 ± 2% of wet weight, respectively). Significant variables associated with a specific category were identified based on leverage/squared prediction error plots. Our analyses revealed that PC 36:6, 32:3, 34:4, 32:2, 31:3, 34:6, 33:5, 31:2, 37:6, 38:2, 40:5, 38:3, 35:6, 37:0, and PE 34:4 and 34:3 were specific for high liver lipid content (Figure 4a). Phosphatidylcholine 32:3, 35:2, 38:1, 35:5, 33:1, 37:2, 37:0, 35:0, 33:0, 37:5, 33:2, 36:4, 39:2, PE 39:0 and 45:4, LPC 30:2 and 30:1, TAG 46:2, and PG 38:4 were specific for high total FAAUC (Figure 4b). Phosphatidylglycerol 36:4, 39:0, 38:4, 38:6, PC 37:5, 32:3, 35:5, 32:0, 31:3, 32:4, 30:2, DAG 40:4, 36:0, 30:2, LPC 22:5, 15:0, 18:0, and PE 37:6 and 34:3 were specific for high BHBAAUC (Figure 4c). Notably, PC 32:3 was specific for high total FAAUC, BHBAAUC, and high liver lipid cows. 

## 3. Discussion

The animals in our study belonged to a cohort of dairy cows in a commercial dairy herd, and we consider them to be representative of high-producing cattle in the Northeastern United States. The dramatic losses in BW and BCS observed in our study were accompanied by suppressed DMI and elevated circulating total FA and BHBA concentrations, which are routinely observed in periparturient dairy cows [19] and reflect the expected metabolic adaptations associated with the onset of lactation [20,21]. Maternal adaptations, such as the dramatic reduction in insulin action, may result in accelerated lipolysis that could predispose cows to enhanced risk for hyperketonemia, fatty liver, and other postpartum disorders. Postpartum cows in this cohort exhibited total FA and BHBA concentrations postpartum, which categorizes them at increased risk for compromised health status postpartum as defined by Ospina et al. [9,10]. Fatty liver, as defined on the basis of liver TAG or total lipids, was observed to be mild prepartum and moderate postpartum, although several animals developed severe fatty liver as defined by Bobe et al. [22]. Of importance to classifying the severity of liver lipid accumulation, total lipid (up to 450 mg/g of wet liver) is highly related to hepatic TAG concentration (2-slope broken-line model; [23]). Overall, our analysis captured the relationships of circulating lipid metabolites against the three major factors currently known to define animals at increased risk for peripartal disease, namely total FA, BHBA, and liver lipid content. These links are relevant because of the known relationship that exists between dysfunctional lipid metabolism and the onset of metabolic disorders in dairy cows [24].

Our analyses revealed the extensive remodeling of the plasma lipidome in peripartal dairy cows, and highlighted the prominence of changes in neutral lipids (e.g., TAG and MADAG) and phospholipids (e.g., LPC, PC, LPE, and PE). Of the several biomarkers that specifically distinguish between days (e.g., reduced TAG 38:2 and MADAG 54:1), some were comparable to those reported by other research groups to distinguish between pre- and postpartum in dairy cows (e.g., increased PC 34:2 and 36:2; [8]). Overall, our analyses confirmed the potential of previously described lipid classes for the identification of peripartal metabolic dysfunction [7,16,25], and also revealed novel biomarkers (e.g., MADAG) whose significance is still poorly understood. 

Changes in circulating TAG were of interest because of their involvement in the pathogenesis of fatty liver, where they accumulate and may disrupt hepatic function [23]. Increased lipid accumulation in the liver (i.e., increased liver TAG) may correspond with a reduction in circulating TAG, indicating a limited capacity for the export of these lipids into the blood. Interest in the FA composition of plasma TAG is also warranted because of their potential role in lipoprotein metabolism (i.e., assembly and secretion; [26]). We observed that plasma concentrations of saturated and monounsaturated TAG were negatively correlated with the temporal advance of the peripartum. Specifically, as cows progressed through lactation, mono- and polyunsaturated TAG (e.g., TAG 60:1, 58:2) and saturated TAG (e.g., TAG 60:0) were consistently and sharply reduced. The significance of TAG FA composition as a potentially limiting factor for lipoprotein secretion requires further investigation.

Our lipidomic investigation also revealed a novel lipid class, MADAG, not yet considered in dairy cow metabolism. Monoalkyl-diacylglycerols are neutral lipids that can be synthesized in hepatic peroxisomes and aggregate with TAG within hepatic lipid droplets [27]. The high degree of concordance in the pattern of changes observed for TAG and MADAG is in agreement with the fact that they are mostly synthesized through the same pathways by the action of acyl transferases [28]. Furthermore, a similar pattern of TAG remodeling was observed for saturated and unsaturated MADAG. Although the metabolic roles of MADAG remain largely uncharacterized, it is speculated that these neutral lipids may serve as precursors for the synthesis of ether phospholipids, a possibility supported by the identification of a similar alkyl/acyl composition in MADAG and PC [27]. Based on their apparent importance as components of lipid droplets and as precursors of PC, future research should explore the possible involvement of these lipids on hepatic PC synthesis, hepatic lipoprotein synthesis, and the development of fatty liver. 

In mammals, PC comprises the majority of the VLDL monolayer following PE and LPC [18]. Reduced hepatic PC concentrations are likely to limit VLDL export [29,30,31] and trigger TAG accumulation in the liver (Bobe et al., 2004). Ruminants in particular have an inherently low capacity to remove TAG from the liver in the form of VLDL [32,33], while it is known that excessive hepatic FA uptake further compromises liver lipid export via the induction of ER stress and the reduction in apoliprotein B100 synthesis [26]. In agreement with our findings, Imhasly et al. [16] reported reduced plasma TAG species with different chain length and saturation, along with concurrent elevations in plasma PC postpartum. We observed modest correlations between PC and TAG acyl moieties in plasma, although the direction of this association (positive or negative) was dissimilar across acyl moieties. Similar to PC, PE are required for VLDL assembly and secretion [34,35,36], although the requirements for these phospholipids have not been yet defined in cows. Despite the gradual rise in circulating PC and PE as lactation progressed, circulating TAG remained low. This may be a reflection of limited hepatic TAG exports or enhanced clearance of TAG by the mammary gland.

It is possible that lipoprotein assembly may be dependent on the FA composition of hepatic PC and PE, with some specific acyl moieties being more physiologically relevant than others. In agreement with this possibility, we observed the postpartum depletion of specific polyunsaturated PC and PE (e.g., PC 36:6, PC 34:6, PC 32:3, and PE 34:4), molecular species which served as plasma biomarkers of hepatic lipid accumulation. A salient observation about these biomarkers is their mostly polyunsaturated nature, notably containing very long-chain FA such as docosahexaenoic acid (DHA; 22:6). The biomarker potential of molecular PC species such as these has been independently observed in peripartal dairy cows [37], where hepatic phospholipids such as PC 36:6 and PE 36:6 were identified as predictors of liver lipid accumulation in the livers of overweight postpartum dairy cows exhibiting accelerated lipolysis and reduced insulin sensitivity. Low 36:6 PC levels could be the result of reduced flux via the PEMT pathway in the liver [38], which uses long-chain PUFA, such as 20:4 and 22:6, for PC synthesis [39]. The potential relevance of these findings is highlighted by studies in non-ruminants, where DHA-containing phospholipids exert anti-steatotic and insulin-sensitizing effects in obese mice [40]. Future work should focus on unveiling the potential of targeted nutritional approaches using omega-3 PUFA in the prevention or alleviation of fatty liver in dairy cows. 

Beyond the warranted focus on omega-3 FA, our results also suggest that other phospholipid biomarkers may be of interest in the context of peripartal metabolic disease. For example, PC 32:3 was identified as a biomarker for increased total FA and BHBA in plasma, as well as a marker of liver lipid accumulation. The robustness of this biomarker is further underpinned by the independent identification of this exact PC moiety in overweight cows exhibiting moderately high fatty liver during postpartum [37]. The relevance and usefulness of these biomarkers will require further validation, and future focus will need to be placed on understanding how and whether lipid-focused nutritional management can impact PC synthesis and the propensity for the development of peripartal disease.

The increased influx of circulating FA into the liver may trigger the synthesis and accumulation of lipotoxic lipids that inhibit insulin signaling in both ruminants and non-ruminants (e.g., ceramides, DAG, and LPC; [41,42]). Lehmann et al. [43] identified LPC 16:0 as a central lipid involved in fatty liver-induced insulin resistance in humans. Similarly, DAG have the ability to induce cellular insulin resistance [44], and have been proposed as a potential biomarker of insulin resistance in humans [45]. In agreement with this, plasma concentrations of total DAG increased after parturition, a period characterized by reduced insulin sensitivity in dairy cows. The ability of LPC or DAG to promote insulin resistance in early-lactation dairy cows is uncertain. 

Our research group has recently started to explore the role of LPC as immunomodulators in dairy cattle. In lactating dairy cattle, circulating LPC concentrations decrease dramatically immediately following an endotoxin challenge (in review). This is intriguing considering that low circulating LPC concentrations are associated with the development of sepsis in humans [46]. In the present study, circulating LPC concentrations were lowest at parturition. This may be a concern considering that immune suppression is a recognized feature of the periparturient period [47]. The possibility exists that low LPC status during the transition period predisposes dairy cattle to infection; however, the mechanism of action is uncertain.

## 4. Materials and Methods

### 4.1. Experimental Design

Experimental procedures were approved by the Institutional Animal Care and Use Committee at West Virginia University (Morgantown). Twenty-seven non-lactating pregnant Holstein cows were randomly enrolled in a study completed at DoVan Farms, a 700-Holstein cow commercial dairy farm (Berlin, PA). Cows were enrolled at d −32 relative to expected parturition, and selected for a body condition score (BCS) between 3.0 and 3.75 (average 3.48 ± 0.10 units; [48]) and lactation number (second or third lactation; average 2.04 ± 0.69 lactations). Previous milk yield for all cows was 10,838 ± 2434 kg of 305 d mature-equivalent yield. Gestation and lactation diets were offered as a total mixed ration (TMR) twice daily (09:00 and 18:00 h) and daily orts were collected before the morning feeding and maintained between 5 and 10%. Cows received diets supplemented with or without 22 g/d of methionine, 10 g/d of choline chloride, 3 g/d of betaine, 96 mg/d of riboflavin, and 1.4 mg/d of vitamin B12; MecoVit, Vetagro S.p.A., Reggio Emilia, Italy as described previously by Zang et al. [49]. Cows were milked twice daily from d 1 through 14 of lactation, and milk yields were recorded daily. Cows were weighed to record body weight (BW), and BCS was assessed by 3 trained investigators weekly. 

Dietary forages and concentrates were sampled weekly and composited monthly. Blood samples (~10 mL) were collected from the coccygeal vein into Vacutainer tubes (Becton Dickinson and Co., Franklin Lakes, NJ, USA) containing EDTA before morning feeding on d −28 (baseline measurements before treatment), −21, and −14, daily from d −7 through 7, and on d 10, 12, and 14, relative to expected or actual calving. For plasma collection, tubes with EDTA were placed on ice for 30 min until centrifugation at 3400× *g* for 10 min at 4 °C. Liver biopsies were conducted on d −28 (baseline), 5, and 14 relative to expected or actual calving, as described by Jorritsma et al. [50]. In brief, the 11th intercostal space was clipped, sanitized with iodine scrub, and anesthetized using 2% lidocaine HCl (Vedco Inc., Saint Joseph, MO, USA). A fabricated trocar was inserted through a small incision to collect approximately 1 g of liver tissue. Separated plasma and biopsied liver tissue were snap-frozen in liquid nitrogen and then stored at −80 °C until further analysis.

### 4.2. Experimental Analyses

Plasma samples were analyzed for total FA, BHB, glucose, insulin, total TAG, and total and free cholesterol. Plasma total FA, BHB, glucose, total TAG, and total and free cholesterol concentrations were measured by enzymatic methods using commercial kits (#999–34691, #995–34791, #991–34891, #993–35191, HR Series nonesterified FA-HR (2); #417–73501, #413–73601, Autokit 3-HB; #997–03001, Autokit Glucose; #994–02891, #990–02991, L-Type TAG M; #439–17501, cholesterol E; and #435–35801, free cholesterol E, respectively; Wako Chemicals USA Inc., Richmond, VA, USA). Plasma insulin concentrations were measured by ELISA using a commercial kit (#10–1201–01, Mercodia Bovine Insulin; Mercodia AB, Uppsala, Sweden). Liver samples were used to determine the percentage of total lipid in liver as described by Starke et al. [19]. 

#### Lipidomics

For lipidomics, plasma samples were extracted using a modified Bligh and Dyer procedure to obtain a crude lipid fraction. In brief, 60 μL of plasma was gently mixed in a glass vial with 940 mL of distilled deionized H_2_O and 2.9 mL of methanol/dichloromethane (2:0.9, *v/v*) containing 1,3(d5)-dihexadecanoyl2-octadecanoyl-glycerol (D-5 TAG 16:0/18:0/16:0, 0.5 ng/mL) and cholesteryl-d7 palmitate (cholesteryl-d7 ester 16:0, 30 ng/mL), purchased from Avanti Polar Lipids Inc. (Alabaster, AL, USA). To obtain a biphasic mixture, an additional 1 mL of distilled deionized H_2_O and 0.9 mL of dichloromethane were added and vortexed. The resultant mixture was incubated on ice for 30 min and centrifuged (10 min, 3000× *g*, 4 °C) to separate the organic and aqueous phases. The organic phase was removed and stored at −20 °C. Just before analysis, 1 mL of the organic layer was dried using a nitrogen evaporator (Organomation Associates Inc., Berlin, MA, USA) and resuspended in 250 µL of running solvent (dichloromethane:methanol (1:1) containing 5 mM ammonium acetate), and 5 mg/mL of ceramide 17:0 used to track instrument performance. All solvents used were HPLC-grade. Lipid analysis was conducted by MS/MSALL on a TripleTOF 5600 (AB Sciex, Redwood City, CA, USA) timeof-flight mass spectrometer (TOF MS). Samples (50 μL injection volume) were infused by HPLC at a constant flow rate of 5 µL/min using a LC-20AD pump and SIL-20AC XR autosampler (Shimazu, Canby, OR, USA). The mass spectrometer was operated at a mass resolution of 30,000 for time of flight (TOF) MS scan and 15,000 for product ion scan in the high sensitivity mode, and automatically calibrated every 10-sample injections using APCI positive calibration solution delivered via a calibration delivery system (AB SCIEX). Source parameters were optimized and set as follows: source parameters were optimized and set as follows: ion source gases at 15 (GSI) and 20 psi (GS2), curtain gas at 30 psi, temperature at 150 °C, positive ion spray voltage at 5500 V, declustering potential at 80 V, and precursor ion collision energy at 10 V. Each sample was run in duplicate in positive ion mode. An initial TOF MS scan provided an overview of the total lipid content at an accumulation time of 5 s. Precursor ions were selected by sequential 1-thomson mass steps from 200 to 1200 *m*/*z*, and the analytes in each 1-thomson step were introduced into the collision chamber. Fragments were produced by collision-induced dissociation and identified by TOF with a scan range of 100–1200 *m*/*z* (accumulation time of 450 ms). The collision energy for each MS/MS step was 40 V. The TOF MS and MS/MSALL data obtained were post-aligned to internal standards using Analyst TF 1.7 (AB Sciex) with mass error less than 5 ppm. Lipid identifications were validated using a pooled sample that was extracted and sequentially analyzed 8 times. Criteria for the inclusion of lipid analytes for analysis were that MS/MS fragment peaks were present in 7 of the 8 pooled runs and the coefficient of variation for peak identifications was less than 20%. Peak identifications meeting these criteria were then used to develop a targeted method in LipidView (AB Sciex, Concord, ON, Canada). The targeted method was used to identify these pre-validated lipid species in experimental samples using a custom made MatLab script and MultiQuant software (version 3.0, AB Sciex, Concord, ON, Canada). All peak intensities were corrected by their corresponding internal standard, and each sample duplicate was averaged. All lipidomic data are denoted as fold-change relative to d −28 (baseline).

### 4.3. Statistical Analyses

#### 4.3.1. Univariate Data

Data were analyzed using a mixed model with repeated measures in SAS (version 9.3; SAS Institute Inc., Cary, NC, USA), according to the following model:

Y_ijk_ = μ + C_i_ + P_j_ + e_ijk_,

where Y_ijk_ = dependent variable, μ = overall mean, C_i_ = random effect of cow (i = 1 to 27), P_j_ = fixed effect of sampling time (j = −28 to 14, d relative to parturition), and e_ijk_ = residual error. Three common covariance structures (variance components, first-order autoregressive, compound symmetry) for repeated measures analysis were evaluated and the structure with the smallest Akaike’s information criterion coefficient, a measure of model quality, was selected for analysis. Normality of the residuals was checked with normal probability and box plots and homogeneity of variances with plots of residual versus predicted values. Skewed data were natural log-transformed when residuals were not normally distributed, back-transformed and reported in the original scale. Data were considered as outliers and removed from analysis when Studentized residuals were >3.0 or <−3.0 (typically 1 per response variable). All results are expressed as least squares means and their standard errors. Significance was declared at *p* ≤ 0.05 and trends at 0.05 < *p* ≤ 0.15.

#### 4.3.2. Multivariate Analyses

We analyzed lipidomic data using the web server MetaboAnalyst 4.0 (www.metaboanalyst.ca; accessed on 19 April 2016 [51]). Non-filtered data were normalized by the sum method, generalized log-transformed, and auto-scaled. Multivariate analysis of data included random forest (RF) classification, partial least squares discriminant analysis (PLS-DA), ANOVA, and Pearson’s correlation coefficient procedures. Lipid biomarkers of peripartal liver lipid accumulation, circulating FA, and ketones were identified using their leverage and squared prediction errors SPE. In order to obtain leverage/SPE plots, cows were categorized into low or high total FA area under the curve (total FAAUC; d 1–14 postpartum; 4915 ± 1369 vs. 12,501 ± 2761 (μmol/L × 14 d); *n* = 18), β-hydroxybutyrate area under the curve (BHBAAUC; d 1–14 postpartum; 4583 ± 459 vs. 7901 ± 1206 (μmol/L × 14 d); *n* = 18), or liver lipid content (d 5 and 14 postpartum; 5 ± 1 vs. 12 ± 2% of wet weight; *n* = 18). Significance was declared at *p* ≤ 0.05 and false discovery rate (FDR) < 0.05. For visualization purposes, heat maps were generated using generalized log-transformed, normalized and auto-scaled data to showcase the magnitude of fold-change in a color gradient for increased (red) or decreased (blue) relative abundance. 

## 5. Conclusions

We conclude that peripartal dairy cows experience extensive remodeling of the plasma lipidome. Several circulating lipids, such as the PC (e.g., PC 36:6 and PC 32:3), appear to be robust biomarkers for alterations in lipid metabolism associated with excess lipolysis (i.e., high total plasma FA), elevated ketogenesis (i.e., high BHBA), and increased hepatic lipid deposition. Future studies should evaluate the usefulness of these biomarkers in terms of their prognostic and diagnostic value for peripartal diseases, and validate them using independent and larger animal cohorts. Our study also provides mechanistic insights into the roles of phospholipids and their FA composition (e.g., DHA-containing PC), which may guide nutritional strategies aimed at improving liver function and cow health during the peripartum.

## Data Availability

The data presented in this study are contained within the article or supplementary material.

## Supplementary Materials

The following are available online at https://www.mdpi.com/article/10.3390/metabo11050290/s1, Figure S1: Temporal changes across lipids classes during the peripartum; Figure S2: Plasma neutral lipids triacylglycerol (TAG) and monoalkyl-diacylglycerol (MADAG) decrease dramatically during the peripartum; Figure S3: Random Forest classification; Figure S4: Relationships between phosphatidylcholines and triacylglycerols in plasma.
