# Supplementary material for: Characterization of the Plasma Lipidome in Dairy Cattle Transitioning from Gestation to Lactation: Identifying Novel Biomarkers of Metabolic Impairment
Source: Metabolites. 2021 Apr 30;11(5):290. doi: 10.3390/metabo11050290 (PMC8147189; doi:10.3390/metabo11050290)
Supplement: Supplementary file 1 [file metabolites-11-00290-s001.zip › metabolites-1161953-supplementary.pdf]

Supplementary Materials

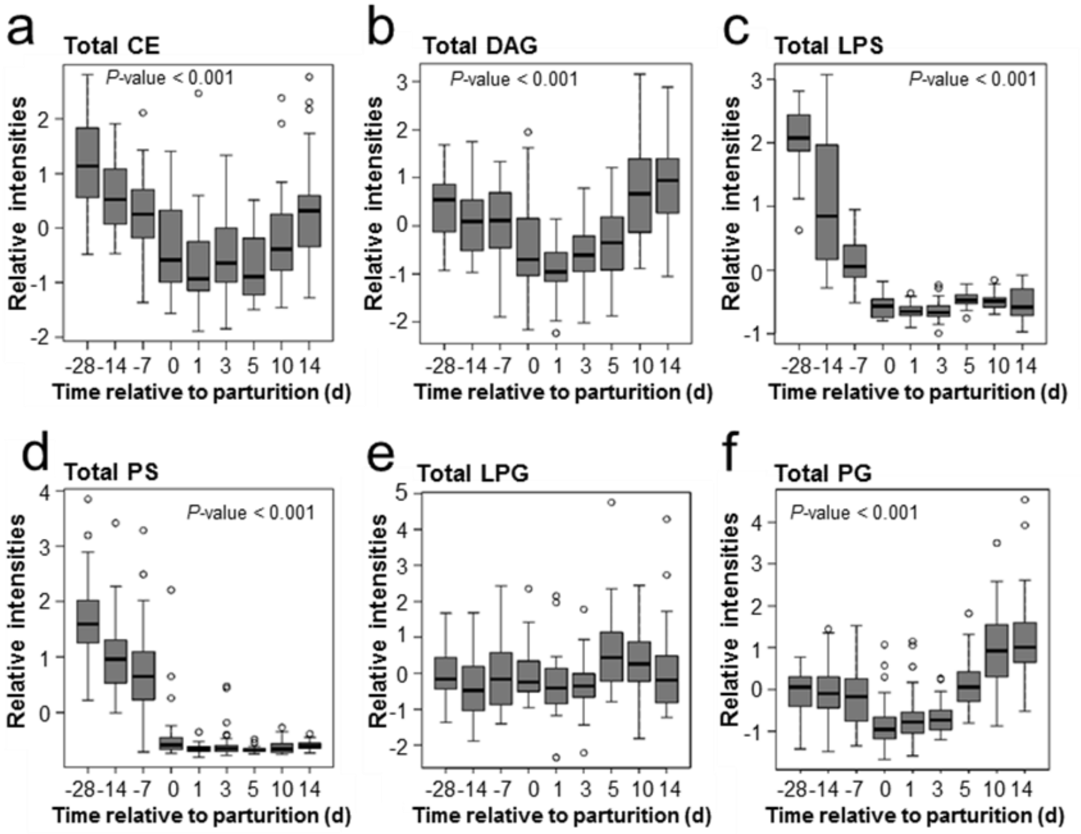

Figure S1. Temporal changes across lipids classes during the peripartum. Normalized, auto-scaled data, reflect the sum of (**a**) cholesterol esters, (**b**) diacylglycerol (DAG), (**c**) lysophosphatidylserine (LPS), (**d**) phosphatidylserine (PS), (**e**) lysophosphatidylglycerol (LPG) and (**f**) phosphatidylglycerol (PG) species measured in plasma. The panels represent data form multiparous Holstein dairy cows (n = 27) at nine time points spanning the peripartum (d −28 to d 14). Lipidomics data were obtained using quadrupole time-of-flight mass spectrometry. Circulating CE, DAG, and PG decrease as cow approaches parturition; circulating LPS, and PS decrease postpartum, and LPG remains unchanged during the peripartum. Data represent plasma from multiparous Holstein dairy cows (n = 27) prior to morning feeding at nine time points spanning the peripartum (d−28 to d14). Lipidomics data were obtained using quadrupole time-of-flight mass spectrometry.

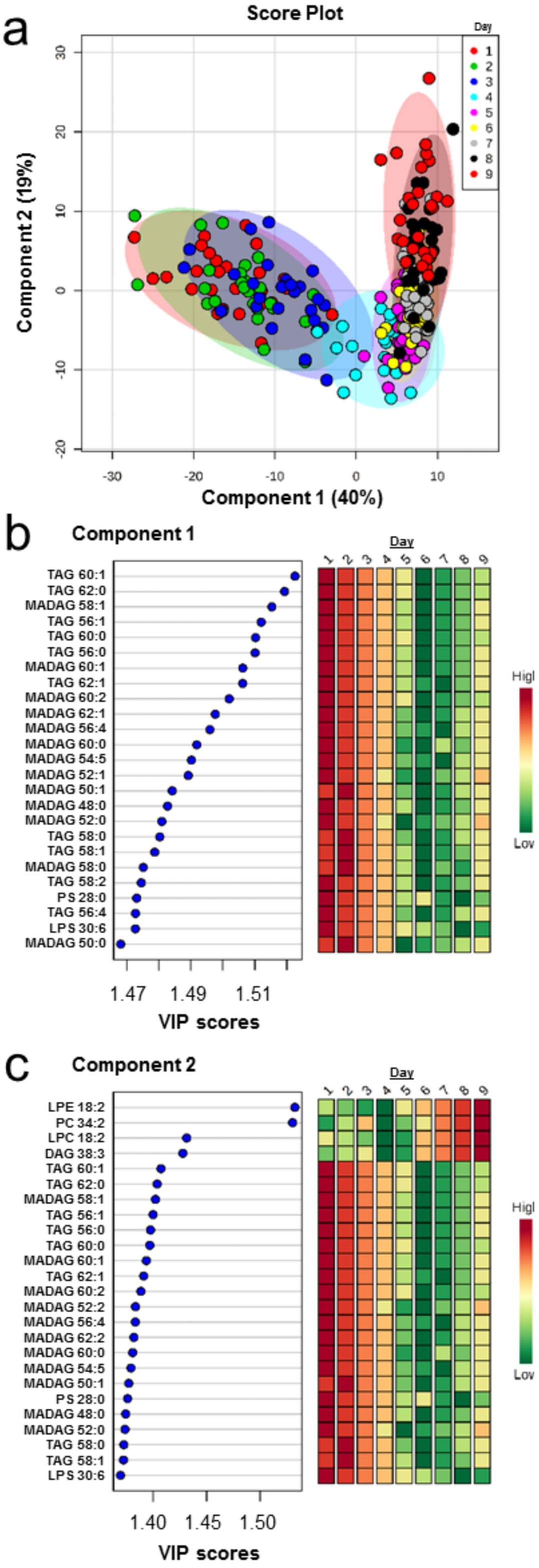

Figure S2. Plasma neutral lipids triacylglycerol (TAG) and monoalkyl-diacylglycerol (MADAG) decrease dramatically during the peripartum. (**a**) Two-dimensional partial least squares discriminant (PLS-DA) score plot, (**b**) VIP scores analysis based on Component 1 of the PLS-DA, and (**c**) VIP scores analysis based on Component 2 of the PLS-DA used to rank the relative contribution of metabolites to the variance between time points. Variance for Component 1 is explained by changes in TAG, MADAG, phosphatidylserine (PS), and lysophosphatidylserine (LPS). Normalized, auto-scaled data is representative of plasma collected from multiparous Holstein dairy cows (n = 27) prior to morning feeding at nine time points spanning the peripartum (d−28 to d14). PLS-DA score plots demonstrate good fitness and high predictability of model with R^2 and Q^2 values ≥ 0.73 and 0.85, respectively. Lipidomics data were obtained using quadrupole time-of-flight mass spectrometry.

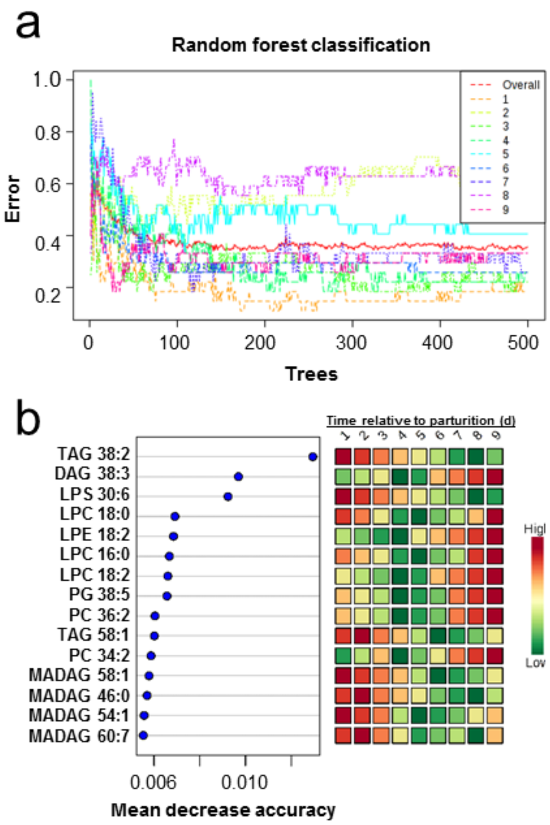

Figure S3. Random Forest classification. Analysis revealed that subjects are distinguishable across nine time points (class error = 0.15). Permutation importance identifies significant metabolites by the Random Forest method. The metabolites are ranked by the mean decrease in classification accuracy when they permuted. Each line represents the error rates for each day. (**a**) Random Forest, (**b**) permutation importance. Normalized, auto-scaled data is representative of plasma collected from multiparous Holstein dairy cows ($n = 27$) prior to morning feeding at nine time points spanning the peripartum (d−28 to d14). Lipidomics data were obtained using quadrupole time-of-flight mass spectrometry.

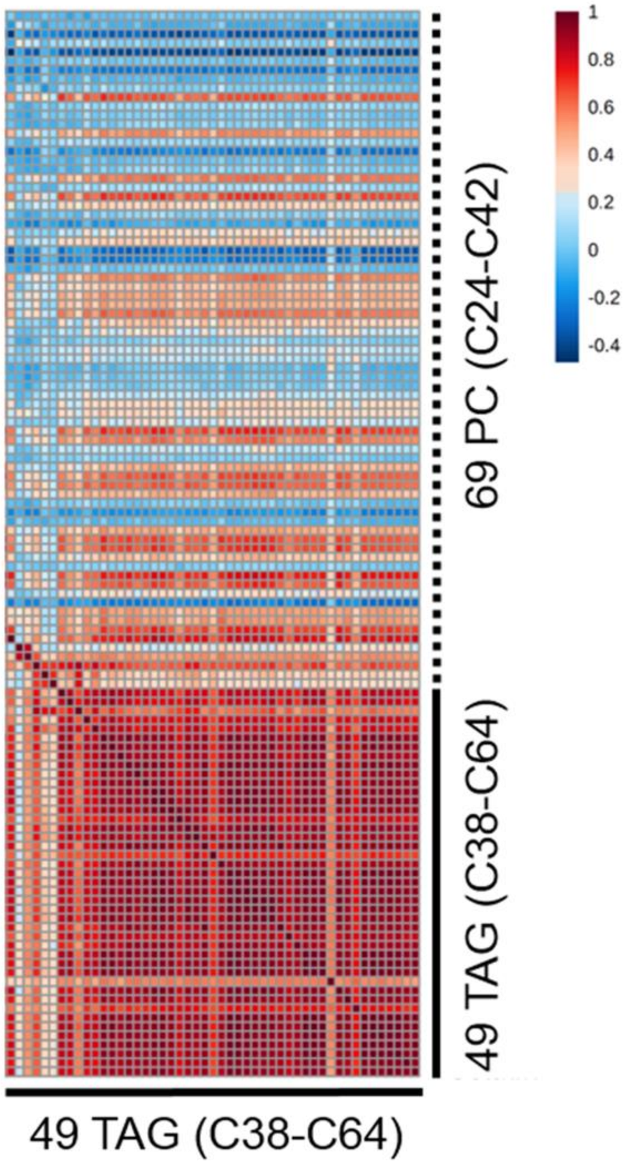

Figure S4. Relationships between phosphatidylchoines and triacylglycerols in plasma. Depending on fatty acyl moiety, plasma phosphatidylcholine (PC) levels are positively or negatively correlated with circulating triacylglycerol (TAG). Pearson's correlation coefficient analysis represents normalized, auto-scaled data collected from plasma of periparturient Holstein dairy cows (*n* = 27) at nine time points spanning the peripartum (d −28 to d 14). Heat map is representative of correlation (*r*)-values. Lipidomics data were obtained using quadrupole time-of-flight mass spectrometry.
